# Alzheimer’s disease transcriptional landscape in ex-vivo human microglia

**DOI:** 10.21203/rs.3.rs-3851590/v1

**Published:** 2024-01-26

**Authors:** Panos Roussos, Roman Kosoy, John Fullard, Jaroslav Bendl, Steven Kleopoulos, Zhiping Shao, Stathis Argyriou, Deepika Mathur, James Vicari, Yixuan Ma, Jack Humphrey, Erica Brophy, Towfique Raj, Pavel Katsel, Georgios Voloudakis, Donghoon Lee, David Bennett, Vahram Haroutunian, Gabriel Hoffman

**Affiliations:** Icahn School of Medicine at Mount Sinai; Icahn School of Medicine at Mount Sinai; Icahn School of Medicine at Mount Sinai; Icahn School of Medicine at Mount Sinai; Icahn School of Medicine at Mount Sinai; Icahn School of Medicine at Mount Sinai; Icahn School of Medicine at Mount Sinai; Icahn School of Medicine at Mount Sinai; Icahn School of Medicine at Mount Sinai; Icahn School of Medicine at Mount Sinai; Icahn School of Medicine at Mount Sinai; Icahn School of Medicine at Mount Sinai; Icahn School of Medicine at Mount Sinai; Icahn School of Medicine at Mount Sinai; Icahn School of Medicine at Mount Sinai; Rush University Medical Center; Mount Sinai and JJ Peters VA Medical Center; Icahn School of Medicine at Mount Sinai

## Abstract

Microglia are resident immune cells of the brain and are implicated in the etiology of Alzheimer’s Disease (AD) and other diseases. Yet the cellular and molecular processes regulating their function throughout the course of the disease are poorly understood. Here, we present the transcriptional landscape of primary microglia from 189 human postmortem brains, including 58 healthy aging individuals and 131 with a range of disease phenotypes, including 63 patients representing the full spectrum of clinical and pathological severity of AD. We identified transcriptional changes associated with multiple AD phenotypes, capturing the severity of dementia and neuropathological lesions. Transcript-level analyses identified additional genes with heterogeneous isoform usage and AD phenotypes. We identified changes in gene-gene coordination in AD, dysregulation of co-expression modules, and disease subtypes with distinct gene expression. Taken together, these data further our understanding of the key role of microglia in AD biology and nominate candidates for therapeutic intervention.

## Introduction

Alzheimer’s disease (AD) is a debilitating illness affecting ~ 57M people worldwide, a number estimated to reach 150M by 2050 ^1^. Despite its high prevalence, AD remains difficult to differentiate from other forms of dementia during life, especially in its advanced stages ^2^. Furthermore, no prognostic biomarkers have been identified that facilitate diagnosis at early stages of the disease, thereby minimizing opportunities for intervention and treatment.

Although primarily associated with neuronal loss, multiple studies have implicated microglia as critical to the etiology of AD ^3^. Microglia are the resident immune cells of the brain, and microglia specific accessible chromatin is enriched in common AD risk variants ^4,5^, providing a road map to the identification of potential therapeutic targets. Study of AD animal models has identified subpopulations of disease associated microglia (DAM) ^6^ while analysis of transcriptional changes associated with lipid droplets inside microglia, an aging-related phenotype, led to the identification of lipid-droplet-accumulating microglia (LDAM) ^7^. However, murine disease signatures have not been robustly observed in human specimens, whereas a largely independent human AD microglia (HAM) signature has been identified, albeit in a study of limited sample size ^8^. An important question relating to AD associated changes is the extent to which they overlap with the biological processes underlying “normal” healthy aging. While HAM signatures suggest accelerated aging processes in human AD ^8, 9^, recent work indicates that AD-associated deterioration and healthy aging can differ significantly ^10^.

To address these gaps in our knowledge, we sought to directly investigate the transcriptional changes associated with AD in *ex-vivo* human microglia, obtained from fresh autopsies (n = 189) from one brain bank and two prospective cohort studies, including individuals with and without age-related neurodegeneration. The availability of detailed clinical and neuropathological data allowed us to evaluate relationships between transcription expression and a variety of AD-relevant phenotypes. The molecular signatures underlying the disease associated changes described here provide an in-depth interrogation of AD biological processes. This allowed us to identify changes in isoform usage not detected at the level of overall gene expression. Hierarchical co-expression analyses identified co-regulated gene modules enriched for distinct AD-associated neurobiological measures and aging, highlighting both unique and shared biological mechanisms. The wealth of the observed transcriptional changes also enabled the exploration of AD heterogeneity, identifying subgroups of patients distinguishable by specific microglial states.

## Results

### Cohort description and disease measures

**Figure 1:**
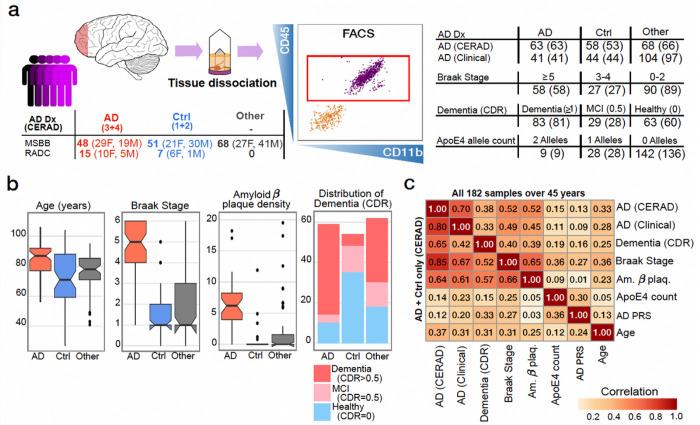
Description of the fresh microglia cohort. **a**) Schematic of the sample processing (left), alongside the distribution of selected AD diagnoses definitions and relevant measures in all 189 initial samples (right). (Sample counts for each measure, ranging from 175 to 189, reflect measure unavailability in certain cases. Brackets indicate counts post-removal of samples from individuals under 45 years; M: Male, F: Female) **b**) Distribution of age, Braak stage, amyloid β density plaque, and CDR dementia rating according to the AD (CERAD) measures within all 189 samples considered for the analyses. Box plots centered on median, bounds defined between the 25th and 75th percentile with minimum and maximum defined as median ± 1.5 × interquartile range (IR), whiskers extending to the lowest/highest value within this range and potential outliers from this shown as dots. **c**) Correlation between variables including AD diagnoses and relevant measures within all 189 samples considered for the downstream analyses (upper right triangle), or only in AD/Control samples (by CERAD) after removing “Other” samples (bottom left triangle).

Microglia were isolated from 193 autopsy donors by fluorescent activated cell sorting (FACS) of viable CD45^+^ cells from fresh (never frozen) dissociated specimens of prefrontal cortex (PFC), corresponding to Brodmann Area 10 (Extended Data Fig. 1, **see** Methods). After quality control filtering of RNA-seq data, 189 donors were retained, including 167 from the Mount Sinai/JJ Peters VA Medical Center Brain Bank (MSBB–Mount Sinai NIH Neurobiobank) and 22 from Rush Alzheimer Disease Center (RADC). Downstream analysis was restricted to the 182 persons over 45 years of age (Fig. 1a, Methods).

Extensive clinical and pathological data captured multiple AD-related phenotypes and enabled high confidence diagnosis of AD. These include: amyloid β plaque density, measured across five brain regions for donors in the MSBB; CERAD score, which is a semi-quantitative metric representing neuritic plaque distribution (1 indicates no neuritic plaque/healthy, 2 is sparse/possible AD, 3 is moderate/probable AD, 4 is frequent/definite AD) ^11^; Braak Stage, indicating tau neurofibrillary tangle (NFT) associated pathology and reflecting disease pathology progression (0 is normal/asymptotic, 1–2 indicate NFT in the locus coeruleus/transentorhinal region, 3–4 indicate progression to limbic regions, and 5–6 indicate widespread NFT) ^12^; and, Clinical Dementia Rating (CDR, 0 is healthy, 0.5 is mild cognitive impairment, and ≥ 1 is dementia) in MSBB and an equivalent physician-curated AD dementia rating in ROSMAP, reflecting behavioral and cognitive symptoms ^13^.

These metrics were used to construct multiple composite phenotypes for downstream analysis (Fig. 1b). The ‘AD (CERAD)’ phenotype indicates AD in donors with CERAD ≥ 2 and includes persons at earlier stages of disease progression. The ‘AD (Clinical)’ phenotype includes donors with CERAD ≥ 3, Braak ≥ 3 and CDR ≥ 1. For these phenotypes, donors with any additional psychiatric or neurodegenerative phenotypes, or any other phenotypes likely to influence the pathophysiology of the brain tissue were considered as ‘Other’ with respect to AD diagnoses. The ‘Dementia (CDR)’ phenotype indicates CDR ≥ 1, and ‘Braak Stage’ is a categorical phenotype separating high values ≥ 5, and low values ≤ 2. ‘amyloid β plaque density’ indicates the mean of this metric across five measured brain regions, and ‘ApoE4 coun’ indicates the number of *ApoE4* risk alleles.

Despite some correlation between these and other AD metrics, these composite phenotypes capture partially independent components of the disease (Fig. 1c). Due to the late age of onset for AD, many of these metrics show correlation with donor age. To further explore this, in addition to the main analyses of the AD phenotypes presented in the paper, which do not include age as a covariate, we also performed sensitivity analyses incorporating age as a covariate or restricting donor age to ≥ 60 years.

### Gene expression signatures of Alzheimer’s Disease

Transcriptionally, the *ex-vivo* microglia examined in this study broadly recapitulate primary microglia signatures observed in two other large publicly available datasets ^14,15^ and to monocytes ^16^, while being distinct from other brain cells, including astrocytes, oligodendrocytes and neurons (Extended Data Fig. 2a).

#### Differential gene expression

**Figure 2:**
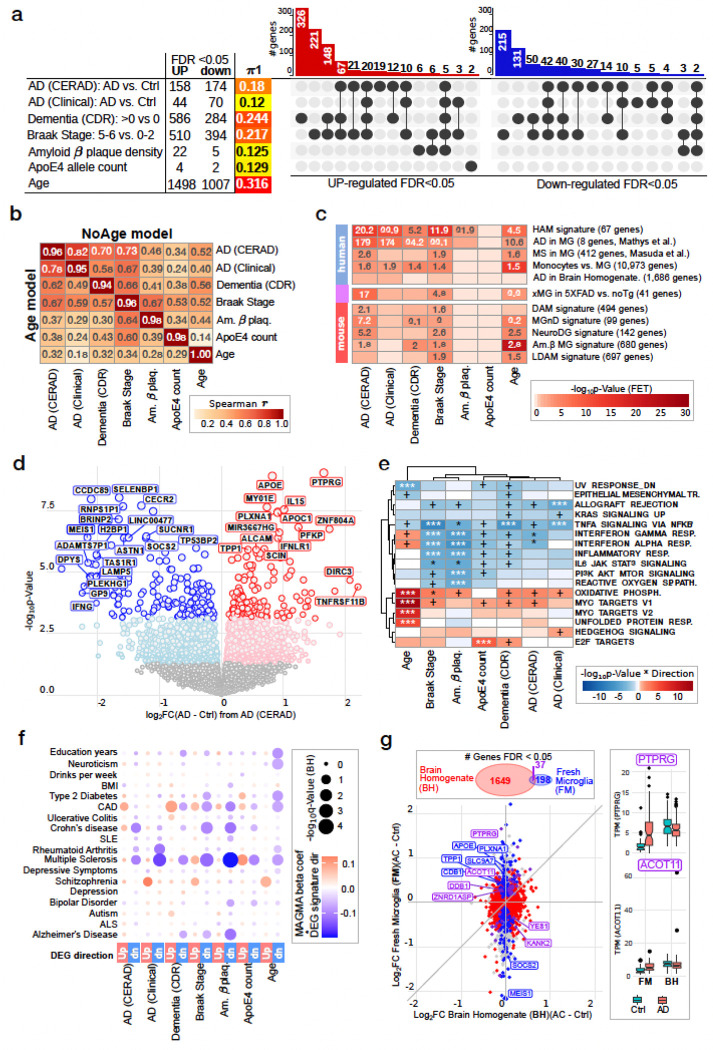
Transcriptional changes associated with Alzheimer’s Disease diagnoses and relevant measures. **a)** The differentially expressed gene signatures for AD relevant measures vary in size and relationship to each other (NoAge model). For the DEG overlap plots Age-derived signatures were removed. **b)** Spearman correlations between the fold change values from the DEG highlight various degrees of the similarity between the associated transcriptional changes. The values in the diagonal represent the correlation between the models with and without Age, above the diagonal are the correlations for analyses without Age, and below with Age. **c)** Comparison between the DEG signatures generated in this study to signatures from previously published studies. Shown are the results for Fisher’s Exact Tests evaluating overlaps between the DEG signatures (FDR≤0.05) from the external analyses to our results (Not including Age in the model): the color and reflects the significance (−log_10_p-value) and the indicated values are Odds Ratio estimates for p-value≤0.05. **d)** Volcano plot for the DEGs from AD Dx based on CERAD score analyses. Top 35 genes are labeled. **e)** Gene set enrichment analyses of genes associated with the AD relevant (Age model) measures using HALLMARK gene signature set from MsigDB ^84^. The color and labels indicate the significance of a parametric enrichment test ^81^ (‘+’ is nominally significant, ‘*’ is FDR≤0.05, and ‘***’ is FDR≤0.01. Top 5 signatures per comparison per direction are included. **f)** Enrichment of genes associated (including Age in the model, p-value≤0.05) with the indicated AD relevant measure for etiologic genetic variants using MAGMA method ^25^. **g**) Comparison of the log_2_ fold change values from differential analyses with AD diagnoses in brain homogenate samples ^27^ versus AD based on CERAD measures in our fresh microglia. The overlap between the significantly associated genes in rain homogenate and fresh microglia are shown in the upper inset amongst the genes used in both studies. TPM counts in AD and cases in brain homogenate (BH) and Fresh Microglia (FM) are shown for *PTPRG* and *ACOT1* genes in the insets.

After removing four outlier samples based on gene expression, transcriptional signatures were generated using mixed linear modeling via the dream software ^17^, including sex, biobank, and technical metrics as covariates (Extended Data Fig. 2b-d, see Materials and Methods). Transcriptional changes associated with aging were corrected for both AD status (“AD (CERAD)” and “Braak Stage”. The genomic features retained for all analyses included 22,097 genes with at least 1 count per million (CPM) in at least 15% of the 189 samples, or 88,749 isoforms with at least 1 transcript per million (TPM) in 15% of the samples (Supplementary Table 1). The number of differentially expressed genes (DEGs) significantly associated with the measures at FDR ≤ 0.05 varies in size, as does the fraction of the genes estimated to be associated with the measures using Storey’s π_1_ statistic ^18^ (Fig. 2a, Supplementary Fig. 2a). The largest signatures for AD-related phenotypes were obtained in comparisons between patients with Dementia (CDR) and Braak score (High vs. Low) (Supplementary Table 1). As expected, the signatures share a large proportion of genes (Fig. 2a), with the effect sizes (log_2_FC) strongly correlated between most comparisons (Fig. 2b). The top genes associated with each of the investigated disease measures tend to be shared across multiple analyses (Extended Data Fig. 3, Extended Data Fig. 4, Extended Data Fig. 5), irrespective of whether Age was included in the model.

#### Effect of Age on DEG signatures

Age-associated transcriptional changes show moderate similarity with AD diagnoses, Dementia, and Braak stage signatures (Spearman r range 0.18–0.34) (Fig. 2b). Inclusion of the age variable in the regression model influences the number of genes significantly associated with AD and other pathology based phenotypes, decreasing the signal in the squared t-statistics by 11.9–23.1%, and increasing the signal for *ApoE4* by 12.5% (Supplementary Fig. 1a). Yet, the effect size estimates and t-statistics from models fit with and without age are highly concordant (Supplementary Fig. 1b-c), indicating that AD signatures are robust and are not dominated by confounding with age.

#### Comparisons of DEG signatures with previously published AD-relevant signatures

To compare the AD-associated DEG signatures identified here to smaller expression signatures from previously published reports, we evaluated the overlap of genes discovered at FDR ≤ 5% (Fig. 2c, Supplementary Fig. 2a) and the correlation between the effect size estimates for human-derived signatures (Supplementary Fig. 2b-c). Of the neurodegeneration-related signatures tested, the most similar to ours were two AD diagnosis signatures: HAM (Human AD microglia) ^8,19,20^, and AD signatures from scRNA-seq data of myeloid brain cells by Mathys *et al.* ^20^. These signatures were significantly enriched for 6 of the 7 AD-related signatures generated here (Fig. 2c). Despite the *ApoE4* genotype being upstream of disease status, the effect size estimates are correlated with these AD signatures (Supplementary Fig. 2b-c).

Transcriptional changes in microglia associated with multiple sclerosis (MS) ^21^, an autoimmune disorder targeting the central nervous system, are enriched in our AD signatures (Fig. 2c), presumably reflecting shared transcriptional changes in the presence of neurodegeneration driven by the two different diseases. Another interesting observation is the highly significant positive correlation between microglia specific genes (when compared to monocytes) ^22^ and genes associated with Dementia (CDR), Braak Stage, amyloid β plaque density, and *ApoE4* counts (Supplementary Fig. 2b-c). Consistent with other reports ^8,19,20^, the signatures associated with neurodegenerative phenotypes in animal models are less representative of human AD associated changes (Fig. 2c, Supplementary Fig. 2a). We do, however, observe significant enrichment between our AD (CERAD) and Braak stage signatures with mouse DAM signatures ^6^ and microglial neurodegenerative phenotype (MGnD) ^23^ signatures (MGnD vs. AD (CERAD): NoAge model: FDR = 4.7x10^−5^, OR = 7.2; Age model: FDR = 0.0093, OR = 11.6).

#### DEG description

Transcriptional changes associated with AD diagnosis based on CERAD score (Fig. 2d, Extended Data Fig. 5a) include the up-regulation of *PTPRG*, *IL15*, *APOE*, and down-regulation of *CECR2*, *SELENBP1*, *ASTN1*, *MEIS1*, and *TNFRSF21*, both with and without Age in the model for our analyses (Extended Data Fig. 6, Supplementary Fig. 3, Supplementary Fig. 4). The same genes have comparable association with Braak stage (Extended Data Fig. 3, Supplementary Table 1), whereas *PTPRG* and *SELENBP1* is less strongly associated with clinical dementia (Log_2_FC AgeModel: for *PTPRG*: AD (CERAD) = 1.64, Braak Stage (High vs. Low) = 1.89, Dementia (CDR) = 0.85; for *SELENBP1*: AD (CERAD) =−1.39, Braak Stage (High vs. Low) =−1.28, Dementia (CDR) =−0.83). The genes with much more significant association with clinical dementia, but not with AD diagnoses, include *SLC46A1* and *LFNG*. Many of the genes associated with AD diagnoses include a number of microglial genes associated with AD in other published studies ^8^.

At the pathway level, inflammatory- and immune-related functions, like interferon α and γ response and TNFα signaling via NF-κB, are down-regulated across multiple AD phenotypes, but not in aging (Fig. 2e, Supplementary Fig. 5a-b). In fact, expression of interferon α and γ response genes tends to increase with age, consistent with aging-related inflammation ^24^. The interferon signaling was particularly strongly down-regulated with Braak stage and amyloid β plaque density, but less so with Dementia (CDR). The reduction in biological annotation with MTOR signaling and reactive oxygen species pathways were much more pronounced for the amyloid β plaque measure, and not observed with AD diagnoses or clinical dementia. Genes up-regulated with AD phenotypes are enriched for oxidative phosphorylation, ribosomal, and ER-related activities, indicating response to stress being potentially associated with advanced neurodegeneration. Genes associated with *ApoE4* allele genotype show only moderate enrichment for immune and inflammatory processes, and no enrichment for oxidative phosphorylation (Fig. 2e, Supplementary Fig. 3).

#### Relationship between genes associated with AD progression and etiology

We examined whether AD transcriptional changes in microglia are enriched for common disease risk variants using MAGMA ^25^ (Supplementary Table 2). Genes that are differentially expressed with AD phenotypes in microglia do not show significant enrichment for AD risk variants (Fig. 2f). This is consistent with the notion that transcriptional changes capture non-genetic and environmental variation, including expression changes being a consequence of disease rather than an upstream cause. Notably, genes down-regulated in AD phenotypes are enriched for risk variants for multiple sclerosis, consistent with disruption of specific immune functions in AD microglia.

### Difference in Brain homogenate vs microglia AD analyses.

Since microglia comprise < 10% of all brain cells ^26^, we investigated whether the AD related changes observed in microglia would be detectable in samples of brain homogenate (BH). We utilized whole brain RNA-seq data from 632 (including 155 AD cases and 86 controls) dorsolateral prefrontal cortex (DLPFC) samples collected as part of ROS/MAP RADC collection ^27^. Out of 1,863 genes significantly (FDR ≤ 0.05) associated with AD diagnosis in the whole brain, 37 were also significantly associated in the microglial AD (CERAD) diagnosis signature (NoAge model: FET p-value = 0.047, OR = 1.4) (Fig. 2g). The magnitude of the AD-associated changes is much higher in isolated fresh microglia (log_2_FC_median_ for FDR ≤ 0.05: Microglia = 0.41; BH = 0.12), reflecting the dilution of less abundant signals in bulk specimens of highly heterogeneous tissues. Surprisingly, of the 37 genes significantly associated with AD in both datasets, six had the opposite directions: *PTPRG*, *ACOT11*, *DDB1* and *ZNRD1ASP* were up-regulated with AD in microglia but down-regulated in the brain homogenate analyses, while *YES1* and *KANK2* had the opposite pattern (Fig. 2g right). Since *PTPRG* is expressed at a low level in microglia relative to the other brain cell types (Control TPM_median_: microglia = 1.25, brain homogenate = 6.47), it is plausible that its apparent reduction in homogenate tissue may be explained by the preferential loss of some cell types with higher *PTPRG* expression in AD. Differential association between *ACOT11* (Acyl-CoA Thioesterase 11) and AD status, due to that gene’s function in lipid metabolism and metabolic activity, suggests a range of metabolic responses across different cell types to AD neurodegeneration.

### Transcript-level expression signatures

#### Concordant and discordant transcript usage analyses

**Figure 3:**
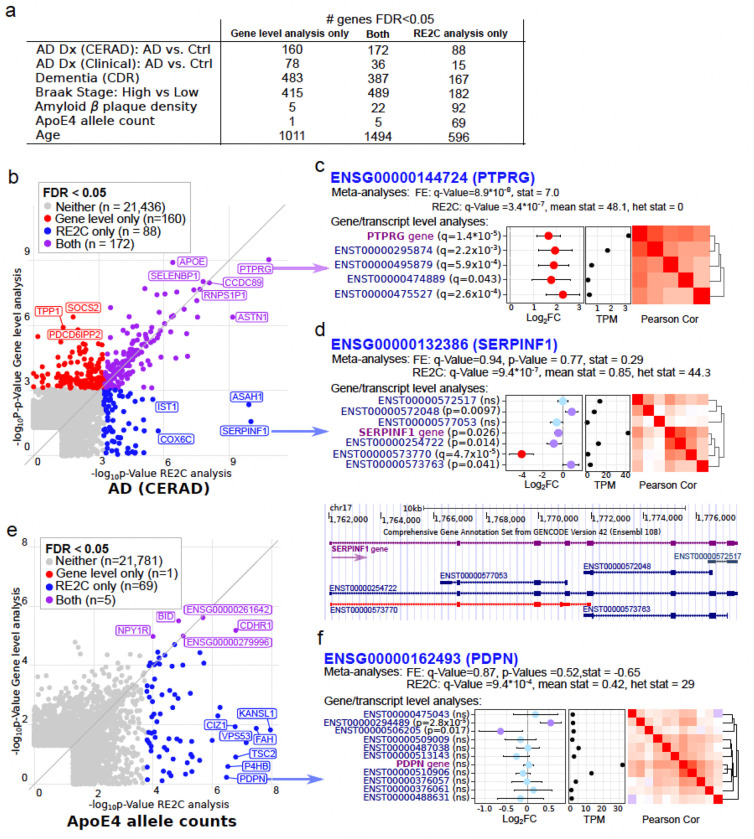
Omnibus analyses of transcript-level data. **a)** Counts of genes identified by differential analysis by a gene-level test, a joint test of effect size and heterogeneity by RE2C, or both. **b)** Comparison of significance for each gene at the gene-level and RE2C omnibus test for AD (CERAD). **c,d)** Effect size, TPM level, correlation structure and statistical tests at the gene-, transcript-, and omnibus-level with AD (CERAD) for **(c)** *PTPRG* and **(d)** *SERPINF1*. Color of points showing effect size indicates significance level at p-value>0.05 (blue), p-value≤0.05 (purple) and FDR≤5% (red). Transcript diagrams are shown for *SERPINF1*. **e)** Comparison of significance for each gene at the gene-level and omnibus test for *ApoE4* counts. **f)** Same plots as above for *PDPN* in relation to *ApoE4* allele counts.

In parallel with gene-level analyses, we also conducted transcript-level analyses including 88,249 transcripts (TPM > 1 in at least 15% of the samples). Yet, due to the low number of counts per transcript and increased multiple testing burden, the number of transcripts associated with AD phenotypes at 5% FDR was smaller compared to gene-level analyses (Supplementary Fig. 1d). Therefore, we used two omnibus meta-analysis methods to evaluate the estimated effect sizes of multiple transcripts per gene while accounting for the correlation structure between transcripts (Supplementary Table 3). The fixed effect (FE) method evaluates whether the mean effect size across transcripts deviates from zero, while the random effect (RE2C) method jointly tests for effect size heterogeneity and mean effect deviation from zero ^28,29^. Importantly, the RE2C test can identify important AD genes missed in summing all reads at the gene-level by identifying effect size heterogeneity across transcripts (Fig. 3a).

#### Detecting genes with transcript-level heterogeneous effects

The RE2C test incorporating *transcript-level* effect size heterogeneity for AD (CERAD) identified 260 at FDR 5%, of which 88 were not identified by the standard gene-level analysis (Fig. 3b). *PTPRG*, the top hit from above, was detected by both the gene-level and transcript-level analyses. Each of the 4 transcripts tested for this gene were significantly associated with AD (CERAD) at 5% FDR. Combining effects of these transcripts with meta-analysis also gave significant findings for the FE (FDR = 9.8x10^−8^) and RE2C (FDR = 3.4x10^−7^) tests driven by highly concordant effects (Fig. 3c).

On the other hand, *SERPINF1* (Serpin Family F protein 1) is not significantly associated with AD (CERAD) at the gene level analysis (p-value = 0.026, NoAge model), or via FE test evaluating effect size concordance (p-value = 0.77), yet is identified by the RE2C test with FDR = 9.4x10^−7^ (Fig. 3d). The entirety of the RE2C signal is driven by heterogenous effect sizes, where a single transcript with the strongest effect showing a very different effect size compared to the other transcripts, and the gene-level results. This transcript, ENST00000573770, generates a truncated product predicted to be targeted by nonsense mediated decay, and is strongly under-expressed in AD patients (FDR = 4.7x10^−5^, log_2_FC=−4.21) (Fig. 3d, Supplementary Fig. 6c-d). Even after excluding this transcript, two additional transcripts (ENST00000572048 and ENST00000254722) that are normally associated with AD (CERAD) on their own also contribute to the heterogeneity signal for this gene (Supplementary Fig. 6d). The down-regulation of *SERPINF1* in AD has been reported previously ^8,30^, yet our data indicate a more complex mechanism operating at the transcript-level in microglia underscored by the fact that this transcript represents an average of 0.62% of *SERPINF1* transcripts in cases and 5.8% in controls.

Of all AD-related phenotypes, heterogeneity analysis of *ApoE* allele 4 count yielded the largest fraction of unique genes not found by the standard gene-level approach, with 74 found by RE2C and 6 by the standard gene-level test (Fig. 3e). One of these top genes with the heterogeneous effects at the transcript-level is *PDPN* (podoplanin), a type-I integral membrane glycoprotein reported as an important component of microglia activation ^31^, ENST00000294489, (p-value = 2.8x10^−5^, FC = 1.4) but does not reach genome-wide significance (Fig. 3f). Yet this transcript, the longest isoform with an alternative transcription start site, drives a heterogeneity signal detected with FDR = 9.4x10^−4^ (Supplementary Fig. 6e-f**)** and represents 8.9% of transcripts in *Apoe4* carriers and 4.6% in the non-carriers.

### Gene-gene relationships influenced by diagnosis

**Figure 4:**
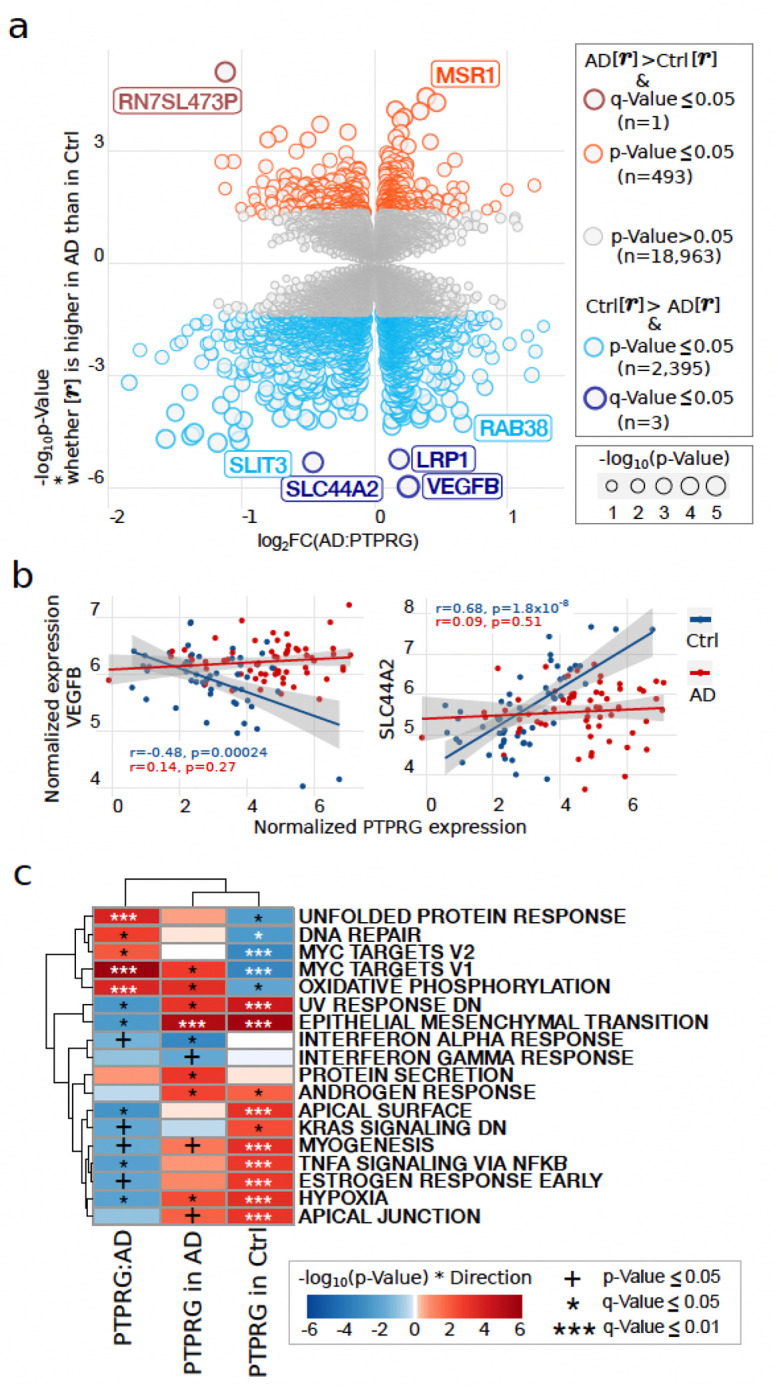
Gene to gene interactions influenced by AD status. **a)** Volcano plot for the genes’ relationships with *PTPRG* differentially affected by AD (CERAD) diagnosis. The color intensity and size indicates the significance level of the *PTPRG*:AD interaction (dark color is FDR≤0.05), light is p-value≤0.05) and color indicates whether the gene is more correlated (absolute magnitude) with *PTPRG* in AD (red) or Control (blue) samples. **b)**. Relationship between the expression levels of the *PTPRG* and the indicated genes in AD (n=63, red dots) and Control (n=53, blue dots) samples in modeled voom-transformed data ^76^. **c)** Enrichment analyses of genes whose relationship with *PTPRG* was significantly altered with AD (CERAD) diagnosis, or in genes whose expression is significantly associated with *PTPRG* in AD or Control samples only using the zenith method and HALLMARK gene signature set from MsigDB ^84^ (+ is nominally significant, * is FDR≤0.05, and *** is FDR≤0.001. Top 5 signatures per comparison per direction are included.

To better understand how Alzheimer’s disease may affect typical gene interactions, we examined differences in gene-gene correlation influenced by the disease status. We focus the analysis on the two top genes (*PTPRG* and *APOE*) based on transcriptional dysregulation in AD (Fig. 2d). We observe that 17.3% (by Storey’s π_1_) of genes in the microglia transcriptome have altered correlation with *PTPRG* changed with AD (CERAD). The genes whose correlation with *PTPRG* is perturbed most significantly include *LRP1*, *VEGFB*, and *SLC44A2*(Fig. 4a, Supplementary Fig. 7a,**b**). *LRP1* (LDL Receptor Related Protein 1) binds over 40 different ligands, including amyloid β and ApoE, and genetic variants in *LRP1* demonstrated synergistic interaction with mutations in tau in an *ApoE4* independent manner ^32,33^. *VEGFB* (Vascular Endothelial Growth Factor B) has been implicated in playing a neuroprotective role in AD by limiting amyloid β-induced apoptosis in murine models ^34,35^ shows a negative correlation with *PTPRG* in controls but increases to have a small positive correlation in AD patients (Fig. 4b). Conversely, *SLC44A2* (Solute Carrier Family 44 / Choline Transporter-Like Protein 2) is important for survival of immune cells via choline metabolism ^36,37^, has a positive correlation with *PTPRG* in controls, decreasing in AD patients (Fig. 4b). Indeed, this decrease in correlation with *PTPRG* in AD patients is shared by 82.9% of genes with p-value ≤ 0.05 and 75% of genes with FDR ≤ 5% in this analysis, indicating a dramatic loss of functional coordination of microglia transcripts with *PTPRG* in AD.

Biologically, processes associated with the genes whose relationship with *PTPRG* is dysregulated in AD include stress response, MYC activity, inflammatory response via TNFα/NFκB, granules and exocytosis, actin activity, NK-cell activity, as well as multiple aspects of the translational machinery (Fig. 4c, Supplementary Fig. 7c). The profound dysregulation in the *PTPRG* interactome with AD is not observed to the same extent with the other AD DEGs. In the case of *APOE*, the number of genes with altered interactome by AD status is smaller (π_1_ = 0.057), with only one, *CAND1*, reaching significant levels (FDR ≤ 0.05) (Supplementary Fig. 8). Highly expressed in immune cells, *CAND1* (Cullin-associated and neddylation-dissociated protein) is a critical regulator of *SCF* (SKP1-CUL1-F) E3-ubiquitin cascade ligase complexes mediating protein degradation ^38^.

### Co-expression modules relevant to AD

#### Network generation and annotation

We utilized MEGENA ^39^ to generate a hierarchical co-expression network with all of the 189 high-quality samples, and identified 922 high-confidence gene modules across eight levels, retaining 306 modules with over 50 genes for further analyses (Supplementary Table 4). These modules were annotated for functional biological processes and could be grouped into 20 larger clusters based on the shared annotation patterns and the associated predominant biological terms for GO signatures (Extended Data Fig. 7).

#### Distribution of disease relevant gene modules

**Figure 5:**
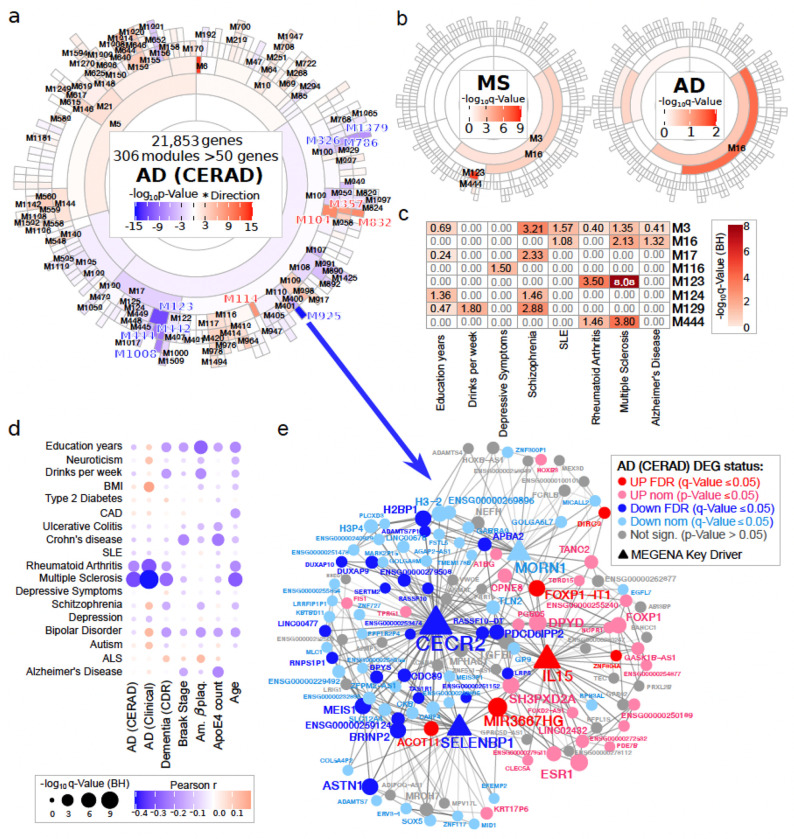
Identification of AD relevant gene modules via co-expression network analyses. **a)** Annotation of gene modules with AD (CERAD) DEG signatures (including Age in the model), with significantly enriched (FDR≤0.05) modules labeled. **b)**Annotation of gene modules with AD and MS GWAS genes (via MAGMA), with significantly enriched (FDR≤0.05) modules labeled. **c)** MAGMA annotation of gene modules across 19 selected GWAS datasets including all GWAS studies and modules with at least one FDR significant enrichment. **d)** Correlation between module annotations for DEG signatures (via zenith) and GWAS genes (via MAGMA). The size of the circle indicates the significance of the Pearson correlation, and color/intensity indicates the direction and r value. **e)** Weighted gene-gene network in M925 module including selected key drivers (*CECR2, MORN1, SELENBP1*, and *IL15*) and all of their first degree neighbors. The weighted edge strength represents strength of the gene-gene relationship, with the size of the node correlating to the number of edges within the entire module. The key drivers are shown as triangles, and color indicates the AD (CERAD) (Age model) DEG status.

To explore co-expression modules relevance to AD, we annotated each gene module with respect to both the generated DEG signatures and GWAS etiologic variants, utilizing zenith and MAGMA methods, respectively. We observed distinct components of the networks enriched for DEG signatures from different AD phenotypes (Fig. 5a, Extended Data Fig. 8, Supplementary Fig. 9). Most of the modules enriched for the genes down-regulated with AD measures were predominantly annotated with immune and inflammatory processes, though the identity of the enriched modules varied greatly between different AD-associated phenotypes. The negative relationship between the gene signatures down-regulated with AD and immune and inflammatory processes, as observed in the direct annotation of DEG signatures (Fig. 2e), is highlighted by the relationship between the DEG enrichments and biological annotations across all modules (Supplementary Fig. 10). Different parts of the microglia network were also enriched for etiologic genes via MAGMA analyses (Fig. 5b–c). Consistent with the observations in direct enrichment of DEG signatures for GWAS genes (Fig. 2f), the annotation for AD diagnoses and Dementia DEG signatures were significantly, but negatively, correlated with MS and rheumatoid arthritis (RA) GWAS genes, and enrichment for AD GWAS was negatively correlated with amyloid β plaque density (Fig. 5d).

#### Gene module subsetting of DEG signatures

A particular utility of co-expression module analyses is subsetting lists of genes, such as DEG signatures, into biologically distinct components. The modules can be further interrogated to identify the key driver genes with the highest degree of connectivity to other members of each interrogated module (Supplementary_Table_4). Focusing on the modules’ enrichment for the AD (CERAD) signature (Fig. 5a, Supplementary Fig. 9), we identify 65 modules significantly enriched for genes up- and 55 down-regulated in AD. Among these, we highlighted a few gene modules of particular relevance (according to the DEG enrichment analyses) to the disease state, M114, M123, M442, M444, M925, and M1379 (Supplementary Results).

Briefly, the module with strongest enrichment for AD genes is M6, composed almost exclusively from non-coding genes (only 1 from 137 module genes is protein coding) (Supplementary Fig. 11). M114 is another module enriched for AD genes, and is anchored by *APOE* key driver, with associated biological annotations, including lipid metabolism and Semaphorin-3A processes (Supplementary Fig. 12). M832, also enriched for AD genes, is anchored by *CD81* and its annotation includes immune cell migration (Supplementary Fig. 13). Among the modules enriched for genes down-regulated with AD, M123, and its child module M444, anchored by *ITK/IKZF3/ETS1*, are significantly enriched for MS and rheumatoid arthritis etiologic genes, and, correspondingly, the modules are strongly enriched for a variety of immune functions (Supplementary Fig. 14). M1379 is primarily associated with purinergic signaling (Supplementary Fig. 15).

A particularly intriguing module is M925, which is enriched for genes down-regulated with all tested AD phenotypes but weaker for the *ApoEA* allele counts signature. The module contains 123 genes, with four key drivers (*CECR2*, *MORN1*, *SELENBP1*, and *IL15*) (Fig. 5e, Supplementary Fig. 16a). Since the MEGENA network was constructed utilizing unsigned similarity (disregarding the correlation direction) the M925 module included a large number of genes both down- (91) and up-regulated (32) with AD, atypical for most of the disease relevant modules usually dominated by genes associated with changes in a single direction regarding a phenotype. Considering M925 genes separately according to their positive or negative association with AD (CERAD), the up-regulated components of the module are enriched for immune processes, whereas the larger, down-regulated component, is enriched for processes involved in homeostasis and morphogenesis (Supplementary Fig. 16b). Delving further into the M925 module Fig. 5e represents the gene-gene interaction plot of the module centered around the four key drivers. Of these, *SELENBP1* (FDR = 6.3x10^−4^, FC = 0.38), *CECR2* (FDR = 0.0012, FC = 0.50) and *MORN1* (p-value = 8.2x10^−4^, FC = 0.75), and most of their first degree neighbors are down-regulated with AD, whereas *IL15* (FDR = 0.0073, FC = 1.7) and its neighbors are up-regulated.

### Stratification of subjects using AD expression signatures

We evaluated whether the gene expression signatures from AD traits could produce biologically informative stratification of subjects. To capture the contribution of multiple AD metrics, we created a combined signature from the top 100 up- and down-regulated genes for each of 5 tested AD phenotypes, plus *ApoE4* allele count and age. In addition, we included genes differentially expressed between patients with Braak score in the middle range (3–4) versus those with high or low Braak stages, with the final signature comprising a total of 1089 genes. We applied nonlinear manifold learning to generate a latent space embedding of 182 samples and performed pseudotime analysis ^40,41^.

**Figure 6:**
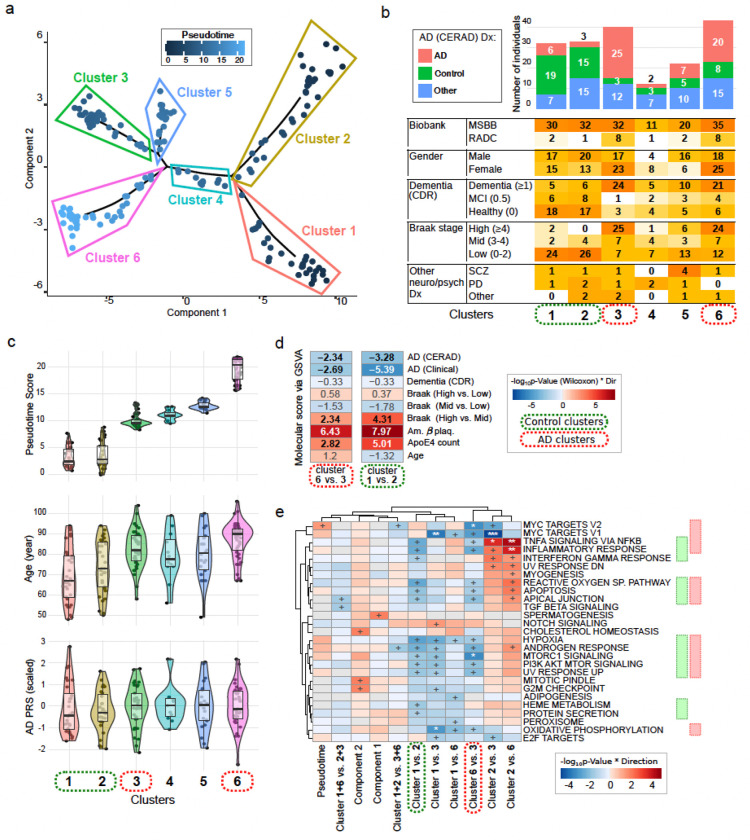
Trajectory analysis of samples utilizing a set of genes from combined DEG signatures distinguishes distinct case and control clusters. **a)** Patient clustering by pseudotime analyses using top 100 up- and down-regulated genes across nine signatures (AD (CERAD), AD (Clinical), CDR. Braak High vs. Low, Braak High. vs Medium, Braak Medium vs. Low, amyloid β plaque density, *ApoE4* counts, and Age), in analyses including Age in the model (1089 genes in total). The color indicates the pseudotime score. **b)** Distribution of the AD (CERAD) Dx and other relevant measures across the identified clusters. **c)** Distribution of Age, and Polygenic Risk Scores for AD ^42^ across the identified clusters. **d)** Comparison of scores from gene set variance analyses between “AD” and “control” clusters. The color intensity and values indicate the −log_10_ p-value from Wilcoxon test comparing the molecular measure scores between “AD” clusters 6 and 3 and “control” clusters 1 and 2. **e)** Enrichment for GO signatures representing biological processes in genes differentially expressed pairwise between the selected four clusters (“Controls” clusters: 1, 2, and “AD” clusters: 3, and 6). The color and labels indicate the significance of parametric enrichment test ^81^ (‘+’ is nominally significant, ‘*’ is FDR≤0.05, ‘**’ is FDR≤0.01 and ‘***’ is FDR≤0.001. Top 5 signatures per comparison per direction are included. Green or red rectangles indicate the biological signatures associated with genes differentially expressed between “control” or “AD” clusters, respectively. For panels **b-e**) “Control” clusters, or comparisons within these, are indicated in dotted green lines: 1, 2. “AD” clusters, or comparisons within these, are indicated in dashed red lines, 3, and 6.

Subjects were grouped into 6 clusters based on a minimum spanning tree ^40,41^ (Fig. 6a). In addition to stratifying subjects based on AD status, the nonlinear embedding further stratifies subjects into two “control” clusters (i.e. 1 and 2) and two “AD” clusters (by AD (CERAD) measure) (i.e. 3 and 6), ignoring “Other” samples (Fig. 6b, Extended Data Fig. 9a). The two “control” clusters share low pseudotime values (mean of 3.75 and 3.31, for clusters 1 and 2, respectively), whereas for “AD”, cluster 6 had higher pseudotime (mean of 19.60) than cluster 3 (mean of 10.12) (Fig. 6c). Subjects in cluster 6 were older than in cluster 3 (age_mean_ (years): cluster 3 = 82, cluster 6 = 90; Wilcoxon test p-value = 0.024), with no observed differences in AD polygenic risk scores (PRS) ^42^ (Fig. 6c). Importantly, the ‘within-control’ and ‘within-AD’ stratification shares with same axis, with distinct molecular states from gene set variance analysis ^43^ based on DEGs from AD diagnoses, (CERAD) and (Clinical), amyloid β plaque density, *ApoE4* allele counts, and Braak stage (High vs. Middle) (Fig. 6d, Extended Data Fig. 10).

Examining some notable AD-associated genes, we do not see significant differences in *PTPRG*, *APOE* or *IL15* expression levels between the “AD” clusters, with (Extended Data Fig. 9b). Yet genes differentially expressed between the two “AD” clusters and the two “controls” clusters are both enriched for immune and stress response related processes, and MTORC signaling (Fig. 6e). MYC target signatures specifically distinguished the two “AD” clusters (stronger in cluster 3), whereas interferon γ responses (higher in cluster 2) distinguished the two “control” clusters. Importantly, the features distinguishing the two “AD” clusters and two “control” clusters between each other are largely similar, both at the level of molecular signatures (Fig. 6d,e) and biological functions. Altogether, this analysis demonstrates that molecular signatures associated with multiple AD relevant measures better captures heterogeneity in both AD patient and healthily aged individuals.

## Discussion

While genetic studies have highlighted the central role of microglia in the molecular etiology of AD ^42,44^, understanding the functional expression changes associated with disease state offers direct insight into AD biology. Large-scale studies of the molecular changes associated with AD in postmortem human brains have used tissue homogenate ^45^ where the contributions of cell-type specific changes can be obscured. Since microglia comprise < 10% of all brain cells ^26^, they are poorly represented in brain homogenate resulting in low power to detect cell type specific molecular changes associated with AD.

To address these limitations, we generated RNA-seq data from purified *ex-vivo* microglia samples covering the full spectrum of AD clinical and neuropathological severity. We utilized fresh tissue from two brain biobanks (Mount Sinai Brain Bank and ROS/MAP) that are heavily utilized in neurobiological studies of AD ^27,45^. We identified differentially expressed genes associated with multiple and partially independent AD-related phenotypes, including amyloid β plaque density, tau-tangle distribution, *ApoE4* allele counts, clinical dementia rating and AD diagnosis at two stringency levels (CERAD only, and a combination of CERAD, Braak stage, and CDR). These gene expression signatures are consistent with previous smaller scale efforts in human microglia ^8,20^, but are markedly different from expression signatures in brain homogenate ^27^. This underscores the importance of the cell type specific biology of AD.

Recent work demonstrates that baseline gene expression in primary, but not cultured, microglia or peripheral monocytes, is enriched for AD genetic risk variants ^22^. Here we observe that, at the genome-wide level, microglia disease state gene expression signatures are independent of AD genetic risk variants. This is consistent with a model of AD biology where genetic susceptibility impacts microglia function, while gene expression signatures associated with disease largely capture secondary changes. As AD progresses, we observe a loss in normal immune related microglial functions, indicating a role for these processes in advanced AD. This is consistent with prior observations ^46^. The degree to which the contribution of other non-genetic AD risk factors follows the pattern of AD risk genes, or that of disease progression observed here, is not clear. Having identified an association between AD progression and stress related processes, it is plausible that other pathologies associated with increased AD risk, such as cardiovascular disease, stroke, and diabetes, might also result from cell type specific changes similar to those of microglia.

Among our findings, *PTPRG, APOE,* and *IL15*, are significantly up-regulated in AD microglia across the range of phenotypes, whereas genes broadly down-regulated include *CECR2* and *SELENBP2. PTPRG* (protein tyrosine phosphatase receptor type G) is the gene with the strongest statistical support for an association with AD diagnosis. *PTPRG* up-regulation is primarily observed in patients with more advanced measures of plaque and tangle deposition, with the highest levels in patients with Braak stage of 4 and higher, and with CERAD of 4. The genetic neighborhood of *PTPRG* altered with AD status includes key AD risk genes *LRP1* and *VEGF*, and is characterized by signatures indicating increased oxidative phosphorylation and MYC target binding, and by reduced hypoxia response and immune response via TNFα/NFκB. *APOE* is highly significantly up-regulated with AD measures, consistent with prior observations ^47^, but less so with increased amyloid β plaque density. Unlike *PTPRG, APOE* has a more linear relationship with Braak score, and is higher in patients with CERAD 3 and 4. *IL15 is* up-regulated in almost all samples with high CERAD (3–4) or Braak score (3–6), but is lowly expressed in half of the controls.

In addition, we used an omnibus testing approach to identify genes with heterogeneous transcript-level expression changes in AD that were not detected at the gene-level. One such example is *SERPINF1*, a gene that includes a single truncated isoform that is strongly down-regulated in AD, as well as two other more common isoforms that are up-regulated. Such cell type specific transcript-level effects are missed in single cell data using 3’ sequencing, as well as in tissue homogenate where microglia expression signatures are obscured by those from more abundant cell types.

Co-expression network analysis deconstructed disease signatures into biologically distinct structures and identified a number of modules enriched for microglia AD gene expression signatures. One module, M925, is highly enriched for AD DEG signatures (primarily down-regulated), with *CECR2, SELENPB1* and *IL15* being key drivers. In light of the compromised inflammatory processes with AD in microglia, *SELENBP1* (Selenium Binding Protein 1) is a very intriguing gene, as failure to maintain sufficient selenium levels adversely affects immune responses and increases cancer risk ^48–50^. The exact role of *SELENBP1* is not well understood, with reduced levels associated with worse cancer prognosis. In the brain, higher expression is observed in the PFC of schizophrenia patients ^51^, while higher circulating *SELENBP1* protein is an adverse biomarker for both traumatic and acute coronary syndrome ^52,53^. *CECR2* (Cat Eye Syndrome Critical Region Protein 2), an epigenetic regulator, promotes metastasis through up-regulation of multiple metastasis-promoting genes, including *CSF1, MMP2,* and *VEGFA* ^54^. Signaling through *CSFR1* (*CSF1* target) is critical for microglia survival ^55^, *MMP2* is an important mediator of neuroinflammation ^56^, and *VEGFA* production by microglia is important for angiogenesis and cerebrovascular remodeling following brain damage ^57^. Therefore, dysregulation of *CECR2* can influence transcription of genes mediating multiple aspects of microglial function. Due to elevated levels observed in dementia patients ^58^, *IL15* has previously been investigated as a biomarker for AD and frontotemporal dementia (FTD). The potential relevance of *IL15* to AD pathophysiology is difficult to establish as it is generally considered to have a neuroprotective role, and its inability to exert such function in AD patients may be one of the symptoms of AD-associated neurodegeneration ^59^.

Stratifying subjects using a nonlinear latent space embedding followed by trajectory analysis ^40,41^ identified clusters with distinct immune and inflammatory states. We identified 6 such clusters, with two composed predominantly of AD subjects and 2 composed predominantly of neurotypical controls. The AD and control clusters are distinguishable from each other by molecular measures reflecting the transcriptional changes associated with AD diagnoses (higher in cluster 3), as well as amyloid β plaque density and *ApoE4* allele counts (higher in cluster 6), but not clinical dementia, even though patients in cluster 3 are younger than in cluster 6. The transcriptional changes associated with cluster 3 are less associated with advanced age, are potentially more influenced by non-age related contributing factors and may represent a more aggressive AD phenotype. Conversely, the transcriptional state associated with cluster 6 may represent a slower form of AD progression, identifying potential therapeutic directions for mitigating AD progression. While the first axis of variation separates AD from control clusters, the second axis captures shared biological heterogeneity that stratifies subjects within both AD and control clusters. Thus, by using AD-associated transcriptional signatures we can describe the heterogeneity amongst both AD cases and controls, highlighting the diversity of the microglia transcriptional landscape and its relevance to neurodegeneration in the human brain.

The observation of dysregulated inflammation and immune processes in AD is consistent with recently published observations at the single cell level ^60^, presenting the erosion of the microglia epigenetic landscape with a corresponding loss of cell-type identity. The observed loss of basic microglial function in advanced disease states posits the intriguing possibility that the restoration of healthy microglia immune functions may be a means to arrest the neurodegeneration that is characteristic of AD.

## Methods

### Data generation

All brain specimens were obtained through informed consent and/or brain donation programs at the respective organizations. All procedures and research protocols were approved by the corresponding ethical committees of our collaborating institutions. The autopsy brain specimens originated from brain donation programs at Rush University Medical Center/Rush Alzheimer’s Disease Center (RADC) in Chicago, IL and The Mount Sinai Biobank (MSBB) including samples collected from JJ Peters VA Medical Center NIH Brain and Tissue Repository in the Bronx, NY.

### MSBB

Samples were collected at NBTR following similar parameters to MSBB–Mount Sinai NIH Neurobiobank cohort ^61^. Collected autopsies were selected to represent a full spectrum of cognitive and neuropathological disease severity in the absence of discernible non-AD neuropathology. All neuropsychological, diagnostic and autopsy protocols were approved by the Mount Sinai and JJ Peters VA Medical Center Institutional Review Boards, with neuropathological assessments, cognitive, and medical and neurological status determinations performed according to previously published procedures. Postmortem brain tissue was placed in an ice-cooled insulated box and transported from the site of recovery to the Neurobiobank laboratory. A section of the frontal pole (Brodmann area 10) was dissected in the Neurobiobank laboratory, and rinsed in ice-cold sterile saline, placed in pre-cooled (4°C) MACS tissue storage solution (Miltenyi Biotec, cat.# 130-100-008), and immediately refrigerated (4°C).

### ROSMAP

Samples were collected at the RADC in Chicago, IL as part of two prospective studies of aging and dementia: the Religious Orders Study (ROS) ^62,63^ and the Memory and Aging Project (MAP) ^63,64^. At the time of enrollment, were older, without known dementia, and agreed to participate in an annual clinical evaluation and organ donation for research. Participants signed an informed consent and a repository consent and were required to sign an Anatomical Gift Act agreeing to donate their brain and spinal cord at the time of death. Extensive annual neuropsychologic evaluations were collected prior to death, with a structured, quantitative neuropathologic examination conducted at autopsy, described at https://www.radc.rush.edu/docs/var/variables.html. After autopsy, the collected tissue was weighted, placed in ice-cold MACS tissue storage solution (Miltenyi Biotec, cat.# 130-100-008) and shipped overnight at 4°C with priority shipping

### Isolation of microglia from fresh brain specimens

Fresh autopsy tissue specimens from DLPFC (Brodmann Area 10) were placed in tissue preservation buffer (Miltenyi Biotech) and stored at 4°C for ≤ 48hrs before processing using the Adult Brain Dissociation Kit (Miltenyi Biotech cat.# 130107677), according to the manufacturer’s instructions. RNase inhibitors (Takara Bio) were used throughout the cell prep. Following de-myelination (Miltenyi de-myelination kit, Miltenyi Biotech, cat.# 130096733) cells were incubated in antibody (CD45: BD Pharmingen, Clone HI30 and CD11b: BD Pharmingen, Clone ICRF44) at 1:500 for 1 hour in the dark at 4°C with end-over-end rotation. Prior to fluorescence activated cell sorting (FACS), DAPI (Thermoscientific) was added at 1:1000 to facilitate selection of viable cells. Viable (DAPI^−^) CD45^+^CD11b^+^ cells were isolated by FACS using a FACSAria flow cytometer (BD Biosciences). Following FACS, cellular concentration and viability was confirmed using a Countess automated cell counter (Life technologies).

### Clinical Measures

#### Collection and harmonization of clinical, pathological, and demographic metadata

Since the brain tissue specimens were collected from two different sites (MSBB and ROSMAP), the available clinical data varies as a function of source, and we harmonized available clinical, pathological, and demographic metadata (age, sex, and genetic ancestry). Additional information considered in the analyses included technical variables (brain bank, sequencing facility, sequence pooling information, postmortem interval (PMI; measured in minutes), APOE genotype (based on genome-wide genotyping of the samples) to describe each cohort.

#### Measuring AD neuropathology

For analysis comparing donors with pathologic AD, the following variables were used to measure the severity of AD neuropathology. The CERAD scoring scheme for neuritic plaque estimates was harmonized for consistency across multiple brain banks, where the scores range from 1 to 4, with increasing CERAD number corresponding to an increase in AD burden; 1 = no neuritic plaque (normal brain), 2 = sparse (possible AD), 3 = moderate (probable AD), 4 = frequent (definite AD).

Braak AD-staging score measuring progression of neurofibrillary tangle (NFT) neuropathology (Braak & Braak-score, or BBScore). A quantitative measure of the regional patterns of NFT estimates across the brain, where 0 is normal and asymptotic, 1–2 indicate initial stages where NFT begins to appear in the locus coeruleus and the transentorhinal region, 3–4 indicate progression to limbic regions, such as the hippocampus and amygdala, and 5–6 indicate NFT are widespread, affecting multiple cortical regions.

### Clinical Diagnoses

Samples from ROSMAP used consensus summary diagnosis of no cognitive impairment (NCI), mild cognitive impairment (MCI), and dementia and its principal cause, Alzheimer’s dementia ^65–67^. MSBB samples used clinical dementia rating (CDR), which was based on a scale of 0–5; 0 = no dementia,0.5 = questionable dementia (very mild), 1 = mild dementia, 2 = moderate dementia, 3 = severe dementia, 4 = profound dementia, 5 = terminal dementia. After consulting with clinicians, we created a harmonized ordinal variable where dementia is categorized into three levels of cognitive decline, independent of AD diagnosis; 0 = no cognitive impairment, 0.5 = MCI (mild cognitive impairment), and 1 = dementia (including 1–5 from CDR score).

### Clinical-pathologic diagnoses of AD

We evaluated a range of AD diagnosis definitions, and focused on two measures: “AD (CERAD)” and “AD (Clinical)”. The former, “AD (CERAD)”, includes cases with CERAD = 2 or 3, and controls with CERAD = 0. A patient was defined as “Other” in the presence of any of the following: **a)** any diagnosis of psychiatric disorder; **b)** diagnosis of a neurodegenerative disorder other than AD or FTD (Parkinson’s Disease (PD), etc); **c)** systemic autoimmune disease such as MS; **d)** diagnosis of cancer directly affecting brain (i.e. glioblastoma); **e)** sepsis as an indicated cause of death; **f)** indicated severe alcoholism.

“AD (Clinical)” was a stricter AD disease definition combining amyloid β/neuritic plaque, NFT, and cognitive impairment measures. AD diagnosis required satisfying all three of the following conditions, allowing for only one missing entry: CERAD > 1, Braak > 2, MCI/Dementia, and no psychiatric or neurodegenerative diagnosis and limited secondary diagnoses (med_con_sum_bl measure < 2, and cogdx_stroke = 0 from ROSMAP, and the “Other” inclusion parameters for “AD (CERAD)”). Control definition required satisfying all three of the following conditions, allowing for only one missing entry: CERAD = 1, Braak = 0–2, no MCI/Dementia, and no psychiatric or neurodegenerative diagnosis, with limited secondary diagnoses. All other patients were defined as “Others”.

### Genotyping and PRS calculation

Genomic DNA for genotyping was extracted from frozen brain or buffy coat using the QIAamp DNA Mini Kit (Qiagen), according to manufacturer’s instructions. Samples were genotyped using Infinium Psych Chip Array (Illumina) at the Mt Sinai Sequencing Core, with 571,496 SNPs retained after QC. Genotypes were then phased and imputed on the TOPMed Imputation Server (https://imputation.biodatacatalyst.nhlbi.nih.gov), retaining variants with an imputation r^2^ > 0.8. Samples with a mismatch between one’s self-reported and genetically inferred sex, suspected sex chromosome aneuploidies, high relatedness as defined by the KING kinship coefficient (KING > 0.0884), and outlier heterozygosity (+/− 3SD from mean) were removed. Additionally, samples with a sample-level missingness > 0.05 were removed, calculated within a subset of high-quality variants (variant-level missingness ≤ 0.02).

The samples of EUR ancestry, as defined by assignment to the EUR superpopulation described by the 1000 Genomes Project ^68,69^), were isolated using the ellipsoid method. Briefly, sample genotypes were first merged with GRCh38 v2a 1000 Genomes Project data (https://wellcomeopenresearch.org/articles/4-50) ^68,69^ using BCFtools version 1.9 ^70^. PLINK 2.0 ^71^ was then used to calculate the merged genotypes’ principal components (PCs), following consideration of variants with minor allele frequency (MAF) ≥ 0.01, Hardy-Weinberg equilibrium (HWE) p-value ≥ 1x10^− 10^, variant-level missingness ≤ 0.01; regions with high linkage disequilibrium (LD) were removed and LD pruning was performed for the remaining SNPs (window size = 1000kb, step size = 10, r^2^ = 0.2). Finally, the first three PCs of the 1000 Genomes Project EUR superpopulation were used to construct a three-dimensional ellipsoid with a diameter of three standard deviations (calculated from the PCs); samples that fell within this ellipsoid were assigned EUR ancestry.

For the EUR-ancestry samples, identified above, autosomal variants of the sample genotypes with an HWE p-value ≥ 1x10^− 6^, MAF ≥ 0.01 and missingness ≤ 0.02 were retained, and polygenic risk scores (PRS) were calculated using AD GWAS summary statistics ^42^. The PRS-CS-auto method ^72^ was used to apply continuous shrinkage priors to the effect sizes from these summary statistics. A EUR LD reference panel provided by the developers of PRS-CS was utilized (https://github.com/getian107/PRScs), which draws from UK Biobank data ^73^. PRS-CS default settings were used for the calculations: parameter a in the γ-γ prior = 1, parameter b in the γ-γ prior = 0.5, MCMC iterations = 1000, number of burn-in iterations = 500, and thinning of the Markov chain factor = 5. The global shrinkage parameter φ was set using a fully Bayesian determination method. Individual-level PRS were calculated using PLINK 2.0 ^71^.

### RNA-seq processing

RNA was extracted from aliquots of up to 100,000 FACS sorted microglia using the Arcturus PicoPure RNA isolation kit (Applied Biosystems). RNA-sequencing libraries were generated using the SMARTer Stranded Total RNA-seq Kit v2 (Takara Bio USA). Libraries were quantified by Qubit HS DNA kit (Life technologiesWhere available, 100,000 cells were sorted into 1.5 ml low-binding Eppendorf tubes containing Extraction buffer, a component of the PicoPure RNA Extraction kit (Arcturus, cat.# KIT0204), and incubated at 42°C for 30 min while shaking at 850 rpm. Samples were stored at −80°C prior to RNA extraction according to manufacturer’s instructions. This included an RNase-free DNase treatment step (Qiagen, cat.# 79254). Samples were eluted in RNase-free water and stored at −80°C until preparation of RNA-sequencing libraries using the SMARTer Stranded Total RNA-seq Pico Kit v1(Takara Clontech Laboratories, cat.# 635005) or v2, according to manufacturer’s instructions. RNA-seq libraries were quantified by quantitative PCR (KAPA Biosystems, cat.# KK4873) and library fragment sizes estimated using High Sensitivity Tapestation D1000 ScreenTapes (Agilent, cat.# 5067–5584).RNA-seq libraries were subsequently sequenced on NovaSeq 6000 (Illumina) machines yielding 2x50, 2x100, or 2x150 bp paired-end reads) and by quantitative PCR (KAPA Biosystems) before sequencing on the Hi-Seq2500 (Illumina) platform obtaining 2x100 paired-end reads.

The generated RNA-seq libraries were processed by RAPiD, a locally implemented standard pipeline. TRIMMOMATIC was utilized to remove adapters and discard low quality reads. Pair end reads were aligned to the human reference genome GRCh38/hg38 via STAR, implementing WASP module to correct for allelic bias in mapping due to individual patients’ genetic variants for 165 samples with available SNP genotyping data ^74^. The generated BAM files contained splice junction, and transcript pseudo-mapping was done via Kalisto, starting with 235,227 transcripts for 60,535 unique genes. The number of features retained for further analyses after CPM > 1 in at least 15% of samples were as follows: at gene-level analyses, 22,097, and at transcript-level analyses, 88,749 (from unique 21,856 genes). Quality control metrics were generated via Picard (v2.2.4; http://broadinstitute.github.io/picard). Sex identity was validated via expression of *XIST* (female-specific) versus *RPS4Y1* (male-specific) gene. Correct identity of the samples was confirmed by concordance between the genetic variants obtained from RNA-seq with those obtained directly available genotypes, as available.

### Differential expression analysis

Starting from 193 samples from autopsy samples (one CD45^+^CD11b^+^ prefrontal cortical microglia sample per individual), 182 samples were retained for the final analyses, after filtering 4 outliers > 3SD based on inter-sample correlation between the samples’ expression, and removing 7 samples with age < 45 years. Count matrices with 22,097 genes (CPM > 1 in ≥ 15% of samples) were normalized using the Trimmed Mean of M-values (TMM) method ^75^, followed by voom transformation ^76^. Mixed linear modeling utilizing Kenward-Roger approximation via dream function ^17^ in the variancePartition R package ^77^ was used for differential expression analyses. Multiple testing correction was performed using the Benjamini-Hochberg procedure ^78^, and a cutoff of 5% was used to identify differentially expressed genes. Covariates substantially contributing the technically induced variability were identified using a combination of multivariate model selection (extension of Bayesian Information Criterion (BIC) approach to multivariate case (https://github.com/GabrielHoffman/mvIC/) and variance partitioning analysis approaches ^77^. The selected technical measures in the minimal model included the following technical measures: “MEDIAN_CV_COVERAGE”, “PCT_CODING_BASES”, “PCT_INTRONIC_BASES”, “PCT_UTR_BASES”, “PERCENT_DUPLICATION”, “MEDIAN_5PRIME_BIAS”, “MEDIAN_5PRIME_TO_3PRIME_BIAS”, “GC_DROPOUT”, “GC_NC_80_100”, “SECOND_OF_PAIR_PCT_ADAPTER”, “WIDTH_OF_90_PERCENT”, “WIDTH_OF_95_PERCENT”, “WIDTH_OF_99_PERCENT”, as well as biological sex (according to sex-specific expressed genes, *XIST* and *RPS4Y1*). Clinical measures were included in the model as required by the specific comparisons: *ApoE4* count and Age analyses included both AD (CERAD) and Braak Stage (High vs. Low) in order to evaluate disease independent effect. The biobank contributing the sample (“source_location”) was modeled as a random effect. Differential analyses were performed both with and without the inclusion of biological age at the time of the sample collection.

In order to minimize the multiple testing issues, we utilized meta-analysis approaches in forms of omnibus tests. One such approach is implementation of fixed effect (FE) meta-analyses for correlated test statistics ^79^ identifying the genes with the concordant pattern of the transcripts associated with tested phenotype. This approach has been extended to a random effect meta-analysis for correlated test statistics that jointly tests deviation of the mean from zero as well as effect size heterogeneity (RE2C) ^28,29,80^. RE2C can quantify the contribution of effect size heterogeneity to the overall signal and can identify genes for which heterogeneity is the major driver. Both of these methods are incorporated in the remaCor R library (https://CRAN.R-project.org/package=remaCor), and we applied both methods testing for both concordant and heterogeneous contribution of multiple transcripts per gene.

### Functional annotation

Biological annotation of the generated DEG signatures was ascertained using a enrichment analysis methods including the original camera method ^81^, it’s implementation for mixed linear models, zenith (https://bioconductor.org/packages/zenith), and Fisher Exact Test (both at FDR and nominal significance thresholds). Relationship between DEG signatures and disease etiologic genes was evaluated through generalized gene-set analysis of GWAS data via MAGMA (Multi-marker Analysis of GenoMic Annotation) ^25^ across 143 GWAS studies, of which 18 most pertinent were retained for final analyses and plotting (Supplementary Table 2).

### Gene-gene relationship

To conduct an *in-silico* prediction analyses of a gene’s functional contribution to the difference between the evaluated phenotypic states, we evaluated the changes in the gene’s transcriptional neighborhood between the states. In short, we tested for the interaction between the gene’s expression and the measure of interest, with the similar model as used for DEG analyses, including the standard set of covariates and age using dream R library. For example, for the testing of the change in gene-gene relationships for *PTPRG* depending on the AD/control status, the formula was:

~0+AD_dx : PTPRG+Sex_Molecular+age+MEDIAN_CV_COVERAGE+PCT_CODING_BASES+WIDTH_OF_99_PERCENT+GC_DROPOUT+WIDTH_OF_95_PERCENT+WIDTH_OF_90_PERCENT+PCT_INTRONIC_BASES+MEDIAN_5PRIME_BIAS+PERCENT_DUPLICATION+SECOND_OF_PAIR_PCT_ADAPTER+GC_NC_80_100+PCT_UTR_BASES+MEDIAN_5PRIME_TO_3PRIME_BIAS+(1|source_location).

The relationship between the tested genes was investigated by correlation in the expression data residualized for the same covariates as in the model used for the analyses.

### Co-expression network analysis

Functionally coherent structures within the entire microglia transcriptome were identified via hierarchical co-expression networks generated via Multiscale Embedded Gene Co-expression Network Analysis (MEGENA) ^39^ starting with the same 22,097 genes as utilized for DEG analyses in all 189 high quality samples. The starting expression matrix was residualized for the same covariates as in the DEG modeling, but retaining the coefficients from Age, gender, and AD (CERAD). The resulting network contained 922 significant modules (identified as locally coherent clusters while maintaining a globally optimal partition), where a gene can belong to multiple modules across multiple levels of hierarchical structure. Of these, 306 contained over 50 genes, and were retained for all following analyses. The modules were annotated zenith for both the DEG signatures identified here, and for biological pathways and signatures from MSigDB ^82^.

To provide a broad functional category annotation for the 306 larger MEGENA modules we applied the following approach: **a**) grouped GO signatures according to their pattern of module enrichment by applying hierarchical clustering to the 2,032 GO functional categories (from GO biology, GO cellular, and GO molecular signature sets) by the −log_10_p-values from zenith enrichment across the 306 modules, selecting the k = 21 as the minimal cluster number that produces signature clusters containing no more than 25%, and no fewer than 0.2% of all functional signatures, with the cluster size between 8 and 445 signatures, median size 29 (the parameters were selected to give as broad and even distribution of cluster sizes as possible); **b**) each cluster was annotated by the name terms of the included signature (treated as bag of words) significantly enriched in each cluster on the background the name terms across name terms across all GO signatures (using Fisher’s Exact Test), with between 1 to 3 terms manually selected to provide a most descriptive and representative label per signature cluster; **c**) each module was assigned the functional signature cluster containing with the signatures with the most significant enrichment within that module (across all GO signatures), with the numbers of modules per signature cluster ranging between 0 and 50 (one signature cluster did not include the most enriched signature in any of the modules).

Relationship between modules and disease etiologic genes was evaluated through generalized gene-set analysis of GWAS data (MAGMA) ^25^ across 143 GWAS studies, of which 19 most pertinent were retained for final analyses and plotting. Key drivers for the generated DEG signatures were identified as satisfying the following parameters: **a)** the gene is present with the tested DEG signatures (BH ≤ 0.05); **b)** the gene is present within a MEGENA modules significantly enriched for the tested signature, and c) the gene is a key driver of the module as identified by Multiscale Hub Analysis (MHA) within MEGENA pipeline.

### Pseudotime analyses

We adopted the pipeline from Mukherjee *et al.*^40^ to apply a pseudotime approach initially created for single-cell analyses, for identifying a trajectory progression across samples in a bulk RNAseq data. The algorithm implements monocle R package ^83^. Briefly, discriminative dimensionality reduction tree (DDRTree) is a manifold learning algorithm that infers a smooth low-dimensional manifold by reverse graph embedding. The algorithm simultaneously learns a nonlinear projection to a latent space generating a spanning tree, with reverse embedding simultaneously learned from the latent space to the high-dimensional data. Pseudotime score is calculated in the following manner: **a)** identifying a root point on one of the two ends of the maximum diameter path in the tree. **b)** the pseudotime of each point is calculated by projecting it to its closest point on the spanning tree and calculating the geodesic distance to the root point. **c)** assigning samples to branches is done by identifying the branches of the spanning tree followed by assigning samples to the branch on which their projection to the spanning tree lies on.

The original code was obtained from the authors’ github site (https://github.com/Sage-Bionetworks/AMPAD_Lineage/blob/paper_rewrites_1/DLPFC_GenerateMonocleDS_new.R), and applied to our data. The gene signature utilized for the trajectory analysis included the top 100 genes up-regulated and top 100 genes down-regulated in the seven phenotypes utilized throughout the study as well as from the comparisons between patients with intermediate Braak stage (3–4) versus patients with high Braak (5–6) or versus low Braak (0–2) scores. The total number of unique genes across these top 100 gene sets was 1089. The starting expression matrix was residualized for the same covariates as in the DEG modeling, but retaining the coefficients from Age, gender, and AD (CERAD).

The identified clusters were annotated with the available clinical measures, AD PRS score, as well as molecular scores representing the transcriptional state representing individual molecular signatures calculated via Gene Set Variance Analysis (GSVA) ^43^: GSVA scores per comparison were calculated separately using the top 200 UP- and and down-regulated genes, and then a single GSVA score was calculated by subtracting down GSVA signature from UP GSVA signature. We also calculated GSVA scores using different gene inclusion cut-offs (top 100 and top 500 genes), and correlation between GSVA signatures across different cut-offs was > 0.97. To identify transcriptional differences between the identified clusters we applied the differential expression gene analyses pairwise to samples per cluster with the standard covariates including Age, followed by enrichment analyses via zenith method. For functional annotation of pseudotime score, and latent space components DEG analyses were done the above measures as continuous variables.

## Supplementary Material

1

## Data Availability

The RNA-seq dataset, clinical metadata, and analysis outputs are available via the AD Knowledge Portal (https://adknowledgeportal.org). The AD Knowledge Portal is a platform for accessing data, analyses, and tools generated by the Accelerating Medicines Partnership (AMP-AD) Target Discovery Program and other National Institute on Aging (NIA)-supported programs to enable open-science practices and accelerate translational learning. The data, analyses, and tools are shared early in the research cycle without a publication embargo on secondary use. Data is available for general research use according to the following requirements for data access and data attribution (https://adknowledgeportal.org/DataAccess/Instructions). For access to data described in this manuscript see: https://www.synapse.org/#!Synapse:syn53210168
