# Supplementary material for: Alzheimer’s disease transcriptional landscape in ex-vivo human microglia
Source: Res Sq. 2024 Jan 26:rs.3.rs-3851590. Preprint. [Version 1] doi: 10.21203/rs.3.rs-3851590/v1 (PMC10854306; doi:10.21203/rs.3.rs-3851590/v1)
Supplement: 1 [file NIHPPrs3851590v1-supplement-1.pdf]

Supplementary Tables

Supplementary Tables are not available with this version.

Supplementary Table 1:

Differentially Expressed Genes analyses not including Age in the model

Differentially Expressed Genes analyses including Age in the model

Transcript and gene features: transcript/gene distribution before after CPM filtering.

Supplementary Table 2:

GWAS studies utilized in MAGMA analyses

Supplementary Table 3:

Transcript level omnibus test results

Supplementary Table 4:

MEGENA module description:

Module structure

Gene memberships including KD labels

MEGENA module DEG enrichments

MEGENA module MAGMA enrichments

MEGENA module GSEA annotation

MEGENA Functional annotation via signature clusters
